# Insulin‐incubated palladium clusters alleviate Alzheimer's disease‐like phenotypes in a preclinical mouse model

**DOI:** 10.1002/mco2.272

**Published:** 2023-06-17

**Authors:** Shengyang Fu, Congcong Li, Weitao Yang, Huili Chen, Yi Wang, Yingbo Zhu, Jie Zhu, Bingbo Zhang, Xiaohuan Xia, Jialin C. Zheng

**Affiliations:** ^1^ Center for Translational Neurodegeneration and Regenerative Therapy Tongji Hospital Affiliated to Tongji University School of Medicine Shanghai China; ^2^ Shanghai Frontiers Science Center of Nanocatalytic Medicine Tongji University School of Medicine Shanghai China; ^3^ Translational Research Center Shanghai Yangzhi Rehabilitation Hospital affiliated to Tongji University School of Medicine Shanghai China

Dear editor,

Alzheimer's disease (AD) is the most common neurodegenerative disease in the elderly without a cure.^1^ Although the amyloid cascade hypothesis is the mainstream theory of AD pathogenesis for decades, it has been challenged due to unsatisfied outcomes of clinical trials that aim to mitigate the amyloid plaque burden. Emerging evidence has suggested that excessive generation of reactive oxygen species (ROS) including superoxide anion, hydroxyl radical, and H_2_O_2_ is a key process to induce neuroinflammation and neuronal loss in AD, implicating ROS as a promising target for AD therapy.^1^ Generally, there are two common types of ROS scavengers, natural enzymes and antioxidant drugs.^2^, ^3^ However, they have not been applied for AD therapeutics yet due to high cost, low stability, difficulty of recycling, and limited scavenging capacity for multiple types of ROS. Inspiringly, nanozymes, the nanomaterials for mimicking the catalytic properties of natural enzymes, have emerged as excellent substitutes for ROS natural scavengers as they are more stable, durable, and cost‐friendly than natural enzymes and antioxidant drugs.^2^, ^3^ Nanozymes therefore have been reported to facilitate the technological innovations of biomedicine, including the development of nanomaterials with multi‐biofunctions for tissue engineering, neurodegenerative diseases, cancer therapy, and disease diagnosis.^4^ Nanozymes can be further decorated/modified with polymer, protein, or cell membrane to protect their catalytic activities and improve their stability and biocompatibility for biological applications.^3^

Recently, we reported the synthesis of an ultrasmall insulin‐incubated palladium nanozyme Pd@insulin (Pd‐In), via a novel biomimetic synthesis method.^2^ This method utilizes the spatial confinement effect and protein‐mediated biomimetic biomineralization, which is convenient, green, and highly effective. Pd‐based nanozyme was chosen due to its specific electronic structure that mimics the catalytic properties of natural enzymes.^2^ Pd‐based nanozymes has been shown to mimic peroxidase, catalase, and superoxide dismutase activities, which scavenge hydroxyl radical, hydrogen peroxide, and superoxide anion.^2^ Insulin was used as the bio‐template for Pd‐based nanozyme synthesis due to its small molecular weight for excellent confinement effect to synthesize ultrasmall nanoparticles and to confer the blood‐brain‐barrier (BBB) penetration capacity to Pd‐In presumably through receptor‐mediated transcytosis.^5^ Pd‐In have demonstrated outstanding multiple ROS‐eliminating ability in mouse brains post traumatic brain injury and promising therapeutic effects on acute neurofunction impairment and neuroinflammation.^2^ Therefore, it is essential to evaluate the therapeutic effects of Pd‐In on AD, a ROS‐related chronic neurodegenerative disease.

Pd‐In was synthesized by an insulin incubation strategy (Figure 1A). High‐resolution transmission electron microscopy displayed the lattice structure of Pd‐In (Figure 1B). Circular dichroism (CD) spectrum characterized the protein secondary structure of Pd‐In demonstrating α‐helix peaks at 190, 207, and 222 nm (Figure 1C). Dynamic light scattering analysis demonstrated that the average size of Pd‐In was 8.2 nm (Figure 1D). Pd‐In exhibited significant scavenging abilities for superoxide anion generated by the reaction between xanthine and xanthine oxidase, H_2_O_2_, and hydroxyl radical produced by the Fenton reaction (Figure 1E). Pd‐In also significantly reversed Rosup‐induced ROS accumulation within N2a neuronal cells, BV2 microglial cells, and A172 astroglial cells, suggesting excellent intracellular ROS scavenging ability of Pd‐In (Figure 1F–G). We further found that Pd‐In exhibited comparable catalytic activity for multiple ROS in two rounds of reactions, characterizing the enzyme properties of Pd‐In (Figure S1).

**FIGURE 1:**
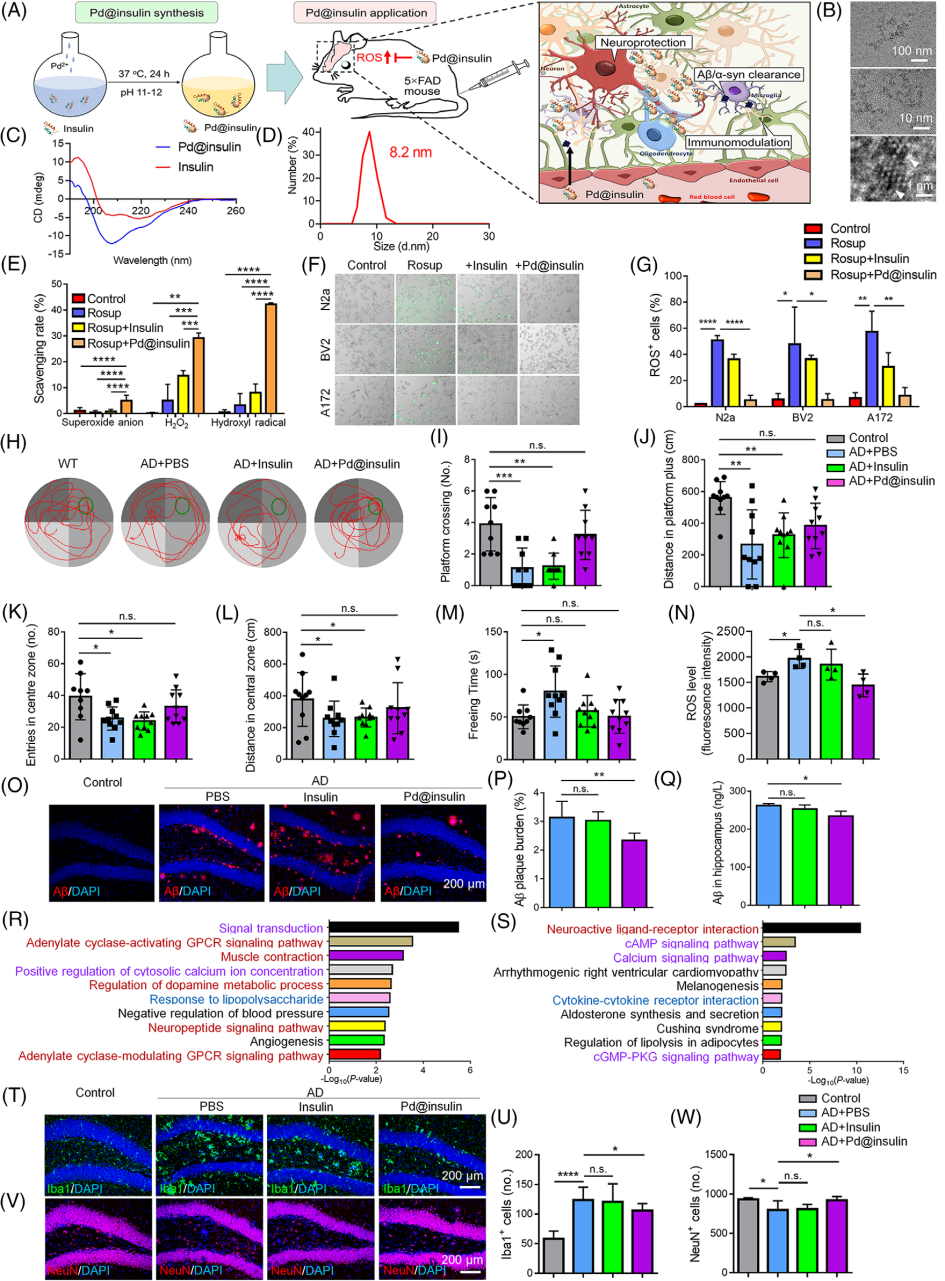
Pd@insulin (Pd‐In) nanoclusters alleviate Alzheimer's disease (AD)‐like phenotypes of 5×FAD mice. (A) Scheme illustration of Pd‐In synthesis and the therapeutic effects of Pd‐In nanoclusters on 5×FAD mice. (B) High‐solution transmission electron microscopy images of Pd‐In (scale bar: 100 nm upper, 10 nm middle, 1 nm lower). (C) CD spectrum characterization of the protein secondary structure of Pd‐In nanoclusters. (D) Size distribution of Pd‐In measured by Dynamic light scatteringDLS. (E) Evaluation of superoxide anion, H_2_O_2_, and hydroxyl radical scavenging ability of Pd‐In nanoclusters (*n* = 5). (F) Intracellular reactive oxygen species (ROS) scavenging ability of Pd‐In evaluation via N2a, BV2, and A172 cells (green fluorescence represents ROS). (G) Quantitative results of ROS^+^ cell proportions (*n* = 3). (H–J) Morris water maze (MWM) tests (*n* = 9–10): (H) Swim paths (red lines) of mice (green circles indicate platforms). (I) Platform crossing numbers, (J) distance travelled by mice in platform quadrants. (K–M) Open field test (OFT) (*n* = 9–10): (K) center zone entry numbers, (l) travel distance in central zone, and (M) freezing time of mice. (N) Hippocampal ROS level by flow cytometry analyses post Pd‐In administration (*n* = 4). (O) Aβ immunoreactivity in the hippocampus. (P) Aβ plaque burden were quantified using ImageJ (*n* = 4). (Q) The levels of Aβ_1‐42_ in the hippocampi were determined by ELISA (*n* = 4). (R and S) The top 10 Gene Ontology (GO) terms (R) and KEGG pathways (S) of differentially expressed genes (DEGs) in comparison between Pd‐In‐ and PBS‐injected mice. Red: neurofunction‐related terms/pathways; blue: neuroinflammation‐related terms/pathways; pink: neuroinflammation‐ and neurofunction‐related terms/pathways. (T) Iba1 immunoreactivity in the hippocampus. (U) Numbers of Iba1^+^ cells were quantified using ImageJ (*n* = 4). (V) NeuN immunoreactivity in the hippocampus. (W) Numbers of NeuN^+^ cells were quantified using ImageJ (*n* = 4). Data are all shown as mean  ±  SD. Statistical analysis was performed by one‐way/two‐way analysis of variance (ANOVA) with a Tukey post hoc test. n.s. denotes no significance. *, **, ***, and **** denote *p* < 0.05, *p* < 0.01, *p* < 0.001, and *p* < 0.0001, respectively.

To determine therapeutic potential of Pd‐In on AD, four‐month‐old APP/PS1 transgenic mice with 5 familial AD mutations (5×FAD) mice were intravenously administrated with either 250 μL Pd‐In (1.2 mg/mL), insulin (1.17 mg/mL), or phosphate‐buffered saline (PBS) every three days. Cy5‐labeled Pd‐In distributed throughout mouse bodies within 15 min (Figure S2). Pd‐In concentration in the blood reached the peak at 15 min, and no obvious remnants were found at 48 h post intravenous injection (Figure S3). Although Pd‐In was mainly accumulated in kidney, lung, and liver (Figure S4A), Cy5 signal could be observed in the brain after PBS perfusion (Figure S4B), suggesting that Pd‐In crossed the BBB and reached brain. The BBB crossing capacity of Pd‐In depends on the insulin receptor‐mediated transcytosis since intraperitoneal injection of 200‐μL insulin receptor inhibitor GSK1838705A (1 mg/kg) significantly reduced Cy5 signal in the brains of Pd‐In‐injected mice (Figure S5). The administration of insulin or Pd‐In did not significantly alter the insulin concentration in mouse brains (Figure S6). No significant difference was observed regarding to blood glucose levels after intravenous administration of Pd‐In presumably due to the alkali‐induced protein degeneration and nanoclusters growth (Figure S7). Cy5‐labeled Pd‐In nanoclusters were observed in Map2^+^ neurons, Iba1^+^ microglia, and Gfap^+^ astrocytes 30 min post administration, indicating the internalization of Pd‐In by brain cells (Figure S8). After one month treatment, a significant increase of platform crossing numbers (Figure 1H–I) and longer distance traveled in the target quadrant (Figure 1J) in the Morris water maze were observed in Pd‐In‐injected 5×FAD mouse group, compared with insulin‐ or PBS‐injected ones. No difference in swimming distance or speed among different groups was observed, indicating comparable gross motor skills across groups (Figure S9). Open field test showed that the administration of Pd‐In nanoclusters increased the entry numbers (Figure 1K) and travel distance (Figure 1L) in the central area, and reduced the freezing times of 5×FAD mice (Figure 1 M) versus insulin‐ or PBS‐injected ones without affecting motor skill (Figure S10). These results suggest long‐term improvements in cognitive function of 5×FAD mice after Pd‐In treatment.

A fluorescent quantitative ROS assay kit confirmed that the excessive ROS accumulation in the AD mouse hippocampus was abrogated by Pd‐In treatment (Figure 1N), indicating positive effects of Pd‐In on scavenging excess ROS in vivo. Moreover, immunohistochemistry results showed significantly reduced amyloid plaque burden in the hippocampi and prefrontal cortices (PFC) of Pd‐In‐injected mice versus insulin‐ or PBS‐injected ones (Figure 1O,P, Figure S11A,B). Enzyme‐linked immunosorbent assay (ELISA) also demonstrated significantly reduced Aβ_1‐42_ levels in the hippocampal and PFC tissue lysates of Pd‐In‐injected mice versus that of insulin‐ or PBS‐injected ones (Figure 1Q, Figure S11C). Hence, our observations demonstrated alleviated Aβ deposition in 5×FAD mouse brains after Pd‐In administration.

To unveil the mechanism underlying Pd‐In‐mediated amelioration of AD‐like phenotypes, transcriptome profiling of hippocampal and PFC tissues collected from all groups of mice was determined through RNA‐sequencing. There were 170 differentially expressed genes (DEGs) (72 up‐regulated and 98 down‐regulated ones) and 120 DEGs (71 up‐regulated and 49 down‐regulated genes) in the hippocampi and PFC of Pd‐In‐injected mice, respectively, compared with PBS‐injected mice (Tables S1‐2). The top 25 DEGs among groups revealed that Pd‐In treatment reversed Aβ‐induced gene expression alterations in the hippocampi and PFC of 5×FAD mice (Figure S12). RNA‐seq results were validated by randomly selecting 4 DEGs and examining their expression patterns via quantitative reverse transcription‐polymerase chain reaction (qRT‐PCR) analysis (Figure S13). The Gene Ontology (GO) and Kyoto Encyclopedia of Genes and Genomes Description (KEGG) analyses suggested the enrichment of total DEGs in comparison of Pd‐In group with PBS group in neuroinflammation and/or neurofunction‐related terms/pathways. (Figure 1R,S, Figure S14). Given the strong associations of identified pathways with ROS in the brain, RNA‐seq analyses suggested that Pd‐In administration mitigate AD‐like phenotypes presumably through mitigating neuroinflammation and enhancing neuroprotection.

Immunohistochemical analysis displayed a significant decrease in the proportions of Iba1^+^ activated microglia and Gfap^+^ activated astrocytes in the hippocampi and PFC of Pd‐In‐treated mice compared with PBS‐injected ones, indicating that Pd‐In inhibits the inflammatory responses of neuroglia of AD mice (Figure 1T,U, Figures S15 and S16). Besides, the numbers of NeuN^+^ neurons also significantly increased in the hippocampi and PFC of Pd‐In‐treated mice compared with PBS‐injected ones, suggesting Pd‐In treatment‐induced suppression of neuronal loss in 5×FAD mouse brains (Figure 1V,W, Figure S17). Moreover, Pd‐In administration also reversed the reduced expression levels of synaptic proteins complexin‐1/2 and Basson in the hippocampi and PFC of 5×FAD mice, demonstrating promising neuroprotective effects of Pd‐In (Figure S18). No effect of Pd‐In treatment on neurogenesis in 5×FAD mouse brain was observed, suggesting negligible neuroregenerative effects of Pd‐In (Figure S19).

In summary, we generated ROS‐scavenging Pd‐In that mitigates AD‐like phenotypes, including cognitive impairment, excessive ROS accumulation, Aβ deposition, neuroinflammation, and neuronal loss. Due to the simple and green synthesis method and its excellent therapeutic effects, Pd‐In can serve as important potential drugs for clinical treatment of AD and other ROS‐related neurological disorders.

## AUTHOR CONTRIBUTIONS

*Conceived and designed the experiments*: Shengyang Fu, Bingbo Zhang, Xiaohuan Xia, and Jialin C. Zheng. *Performed the experiments*: Shengyang Fu, Congcong Li, Huili Chen, and Jie Zhu. *Analyzed the data*: Shengyang Fu, Congcong Li, Weitao Yang, Huili Chen, Yi Wang, Bingbo Zhang, Xiaohuan Xia, and Jialin C. Zheng. *Contributed reagents/materials/analysis tools*: Shengyang Fu, Congcong Li, Weitao Yang, Yi Wang, Yingbo Zhu, Bingbo Zhang, Xiaohuan Xia, and Jialin C. Zheng. *Wrote the paper*: Shengyang Fu and Xiaohuan Xia. All authors have read and approved the final manuscript.

## CONFLICT OF INTEREST STATEMENT

The authors declare they have no conflict of interest.

## FUNDING INFORMATION

National Natural Science Foundation of China (grant numbers: 91949204 and 81830037 to J.C.Z.; 81971145 and 82271477 to X.X.; 81922035 and 81871399 to B.Z.) and Shanghai Sailing Program (grant number: 20YF1437900 to Y.Z.).

## ETHICS STATEMENT

This project was permitted and approved by the Institutional Animal Care and Use Committee of Tongji University School of Medicine (reference number SYXK (HU) 2014‐0026).

## Supporting information

Supporting Information

Supporting Information

Supporting Information

## Data Availability

All data are available from the corresponding author upon request.
