# Supplementary material for: Insulin‐incubated palladium clusters alleviate Alzheimer's disease‐like phenotypes in a preclinical mouse model
Source: MedComm (2020). 2023 Jun 17;4(4):e272. doi: 10.1002/mco2.272 (PMC10276888; doi:10.1002/mco2.272)
Supplement: Supplementary file 3 — Supporting Information [file MCO2-4-e272-s003.docx]

**Supplementary Information for**

**Insulin-incubated palladium clusters alleviate Alzheimer's disease-like phenotypes in a preclinical mouse model**

*Shengyang Fu^1#^, Congcong Li^1#^, Weitao Yang^1,2^, Huili Chen^1^, Yi Wang^3^, Yingbo Zhu^1^, Jie Zhu^1^, Bingbo Zhang^1,2*^, Xiaohuan Xia^1,2^*^*^*, Jialin C. Zheng^1,2*^*

^1^Center for Translational Neurodegeneration and Regenerative Therapy, Tongji Hospital affiliated to Tongji University School of Medicine, Shanghai 200065, China. ^2^Shanghai Frontiers Science Center of Nanocatalytic Medicine, Tongji University School of Medicine, Shanghai 200331, China. ^3^Translational Research Center, Shanghai Yangzhi Rehabilitation Hospital affiliated to Tongji University School of Medicine, Shanghai 201613, China.

^#^These authors contributed equally to this work

*Corresponding authors: Drs. Jialin C. Zheng, Xiaohuan Xia, Bingbo Zhang; Email: [jialinzheng@tongji.edu.cn](mailto:jialinzheng@tongji.edu.cn), xiaohuan_xia1@163.com, bingbozhang@tongji.edu.cn.

**This PDF file includes:**

Materials and Methods

Figures. S1 to S19

Table. S1 to S2

**Materials and Methods**

**Mice**

Four-month-old male 5×FAD and C57BL/6 mice were purchased from Shanghai Model Organisms Center, Inc. All mice were housed and bred at the Comparative Medicine Animal Facilities of Tongji University School of Medicine. All procedures were conducted according to protocols approved by the Institutional Animal Care and Use Committee of Tongji University School of Medicine (reference number SYXK (HU) 2014-0026).

**Synthesis of** **Pd@insulin nanoclusters**

Pd@insulin nanoclusters were prepared using following protocol. Insulin powder (bovine pancreatic, 30 mg) was routinely dissolved in 50 mL deionized water at 37 °C with magnetic stirring, and 25 μL Na_2_PdCl_4_ solution (Sigma-Aldrich, 100 mM) was added gradually. Subsequently, the pH of the solution was adjusted to 10-11 with 1 M NaOH (Sinopharm Chemical Reagent). After 24 h, a pellucid Pd@insulin cluster solution was obtained and dialyzed (Mw = 3 kDa) against ultrapure water. A Pd@insulin cluster powder was obtained by freeze drying.

**Characterization of Pd@insulin nanoclusters**

TEM and high-resolution transmission electron microscopy (HRTEM) were conducted with a JEM-2100 microscope operated at 200 kV and a JEM-2100 microscope equipped with an EDX energy-dispersive spectrometer. Size distribution of Pd@insulin nanoclusters was measured by dynamic light scattering (DLS, Nano-ZS90, Malvern). CD spectrum was conducted with a Thermo Scientific Nicolet iS10 spectrometer in the range of 4000–400 cm^−1^ and a Jasco J-815 spectrophotometer.

**ROS scavenging ability of Pd@insulin nanoclusters**

*In solution*: superoxide anion, hydroxyl radical, and H_2_O_2_ scavenging capacities of Pd@insulin nanoclusters were determined by assay kits (Jiancheng, Nanjing). Superoxide anion generated by the reaction between xanthine and xanthine oxidase, and hydroxyl radical produced by the Fenton reaction. The levels of superoxide anion and hydroxyl radical were measured at a wavelength of 550 nm *via* chromogenic Griess reagent. H_2_O_2_ were detected by ammonium molybdate with an absorbance peak at 405 nm.

*In vitro*: ROS assay kit containing DCFH-DA probe and Rosup (positive control) was used to evaluate ROS scavenging capacity of Pd@insulin *in vitro*. Mouse neuroblastoma cell line (N2a), mouse microglia BV2 cells, and human glioma A172 cells were seeded in 24-well plates, and Rosup was used to induce high levels of ROS *in vitro*. The medium was replaced with Pd@insulin or insulin for 2 h. Afterwards, the cells were washed with serum-free medium thrice, and fresh medium containing 10 μM DCFH-DA probe was added for 30 min. Finally, the cells were washed thrice with serum-free medium for fluorescence imaging.

**Cell culture**

N2a and BV2 cells were cultured in DMEM supplemented with 10% fetal bovine serum and 1% streptomycin and penicillin in a 37 °C incubator. A172 cells were cultured in DMEM/F12 containing the same concentrations of FBS and antibiotics in a 37 °C incubator.

**Analysis of blood glucose levels**

Fresh blood (5 μl) was obtained from the tail of each mouse, and blood glucose levels were determined using a commercial glucometer (Yuyue 580, Jiangsu).

**Pharmacokinetics of Pd@insulin clusters**

The pharmacokinetics of Pd@insulin was determined by measuring the Pd^2+^ ion concentration in mouse blood. At different time points (1 min, 5 min, 15 min, 30 min, 1 h, 2 h, 24, and 48 h), fresh blood (5 μl) was obtained from the tails of Pd@insulin-administrated mice and dissolved in aqua regia for quantification of the Pd^2+^ ion concentration by ICP-MS.

**Behavior tests**

*Morris water maze (MWM)*: MWM was performed as previously described [4]. Briefly, mice were introduced into a circular, water-filled tank which was equally divided into four quadrants. Visual cues were placed around the pool in plain sight of the mouse to flag the submerged platform. Various parameters of mouse movement were recorded, including the time spent in each quadrant of the pool, the time taken to reach the platform (escape latency), and the total distance travelled. For each trial, the mouse was allowed no more than 60 sec to find the submerged platform before they were guided to the platform, removed from water, towel dried, and returned to their cage. Each mouse completed 4 trials per day during the 6-day training phase. One day after the training, the probe test was conducted. The platform was removed and each mouse was given 60 sec to swim in the water. The swimming was videotaped and analyzed by Ethovision XT (Noldus, Netherlands).

*Open field test (OFT)*: Open field test was performed as previously described [6]. Briefly, open field test was performed using an open field apparatus (30 × 30 × 21 cm) with nine virtual quadrants (10 × 10 cm each). Central area is a region consisting of four sub squares marked with red color. Each mouse was put in the center at start of test and permitted to freely explore for 5 min. Number of crossings, time spent in the center and periphery, rearing, fecal pallets, time of immobility, jumping, and efforts made by each mouse to getaway were recorded. The total distance and time as well as the distance travelled and time spent in the center zone (10 cm × 10 cm) were analyzed using the Activity Monitor software (Med Associates, Inc.). The apparatus was cleaned with 70% ethanol between trials.

**Immunofluorescence staining**

Brain sections were fixed in 4% paraformaldehyde, washed three times with PBS, and incubated in permeabilization and blocking buffer (3% BSA, 10% donkey serum, and 1% Triton X-100 in PBS) for 1 h. The brain sections were incubated with primary antibodies including Aβ (mouse, cat# 15126S, CST, 1:400), GFAP (chicken, cat# AB5541, Milipore, 1:500), Iba1 (goat, cat# ab5076, Abcam, 1:100), NeuN (mouse, cat# MAB377, Sigma-Aldrich, 1:500), Basson (Rabbit, cat# 6897 CST, 1:200), DCX (rabbit, cat# 4604, Cell Signaling Technology, 1:800), and Ki67 (rabbit, cat# 9129S, CST, 1:400) overnight at 4 °C. Then, all sections were washed with PBS and incubated with secondary antibody for 1 h. Immunofluorescence was observed under a confocal microscopy (FV3000, Olympus).

**Transcriptome analysis**

Mice were randomly divided into the sham, AD+PBS, AD+Insulin, and TBI+Pd@insulin groups and sacrificed for brain tissue collection after 6 days of treatment. Total RNA was extracted using a mirVana miRNA Isolation Kit (Ambion) following the manufacturer’s protocol. RNA integrity was evaluated using an Agilent 2100 Bioanalyzer (Agilent Technologies, Santa Clara, CA, USA), and samples with an RNA integrity number ≥ 7 were used for subsequent analysis. Libraries were constructed using a TruSeq Stranded mRNA LTSample Prep Kit (Illumina, San Diego, CA, USA) according to the manufacturer’s instructions. These libraries were sequenced on the Illumina sequencing platform (Illumina NovaSeq 6000), and 125-bp/150-bp paired-end reads were generated. Transcriptome sequencing and analysis were conducted by OE Biotech Co Ltd. (Shanghai, China). Raw data (raw reads) were processed using Trimmomatic. Reads containing poly N and low-quality reads were removed to obtain clean reads. Then, the clean reads were mapped to the reference genome using HISAT. The FPKM and read counts value of each transcript (protein-coding) were calculated using bowtie2 and eXpress. DEGs were identified using the DESeq (2012) functions estimateSizeFactors and nbinomTest. A P value < 0.05 and a fold change > 1.50 or fold change < 0.67 were set as the thresholds for significantly different expression. Hierarchical cluster analysis of DEGs was performed to assess transcript expression patterns. GO enrichment analysis and KEGG pathway analysis of the DEGs were performed using R based on hypergeometric distribution.

**Quantitative reverse transcription-Polymerase Chain Reaction (qRT-PCR)**

The mRNAs were isolated from hippocampal and prefrontal cortical tissues using RNeasy mini kit (Qiagen) according to the manufacturer’s instructions. Genomic DNA was removed using DNase I digestion kit (Qiagen), and cDNA was synthesized using OligodT primers with a Transcriptor First Strand cDNA Synthesis Kit (HiScript III All-in-one RT SuperMix Perfect for qRT-PCR, Vazyme). Transcripts were amplified using gene-specific primer (*Gapdh* forward primer: CATGTTCCAGTATGACTCCACTC, reverse primer: GGCCTCACCCCATTTGATGT; *Tlr8* forward primer: GCCAAACAACAGCACCCAAAT, reverse primer: AGGCAACCCAGCAGGTATAGT; *Nlrc4* forward prime: TTGAAGGCGAGTCTGGCAAAG, reverse primer: GGCGCTTCTCAGGTGGATG; *Mpo* forward primer: AGTTGTGCTGAGCTGTATGGA, reverse primer: CGGCTGCTTGAAGTAAAACAGG; *Slc5a11* forward primer: CCACGATGCCAGAATATCTAAGG, reverse primer: GCAAGGACTGCTGAATGAAGA) and SYBR green PCR kit (Qiagen) with the ABI7500 (Applied Biosystems). All qRT-PCR results measured each sample in triplicate and no-template blanks were used for negative controls. Amplification curves and gene expression were normalized to the house-keeping gene *Gapdh*.

**Enzyme-linked immunosorbent assay (ELISA)**

The levels of Aβ_1-42_ in mouse hippocampal and cortical tissue lysates were measured by ELISA kits (cat# KE1266, Immunoway) according to manufacturer’s protocols. Diluted standards and lysates in triplicate were added to the corresponding wells and incubated at room temperature (RT) for 2 h on a microplate shaker. Sample Diluent was used as blank control. 100 μl Streptavidin-HRP was added to each well and incubated at RT for 45 min. 100 μl TMB Substrate Solution was then added to each well and incubated at RT for 30 min. The enzyme reaction in each well was terminated by 100 μl Stop Solution. Absorbance of each well was determined using spectrophotometer DV8200 (Drawell).

**Western blotting**

Brains tissues were homogenized by a homogenizer in the M-PER Protein Extraction Buffer (Pierce) containing a protease inhibitor cocktail (Sigma). Protein concentrations were determined using BCA Protein Assay Kit (Pierce). Proteins (10 μg) from tissue lysates were separated using sodium dodecyl sulfate-polyacrylamide gel electrophoresis (SDS-PAGE). Proteins were electrophoretic transferred onto polyvinyldifluoridene membranes (Millipore and Bio-Rad), and then treated with purified primary antibodies for Complexin-1/2 (Rabbit, 1:1000, CST #28070) or β-actin (mouse, 1:5000, Sigma #A5441) overnight at 4°C followed by a horseradish peroxidase-linked secondary anti-rabbit or anti-mouse antibody (Cell Signaling Technologies, 1:10,000). Antigen-antibody complexes were visualized by Pierce ECL Western Blotting Substrate (Thermo Fisher Scientific). Films were then scanned with a CanonScan 9950F scanner and the acquired images were analyzed on a Macintosh computer using ImageJ program.

**Statistical analyses**

Differences between two independent groups were analyzed by unpaired Student’s t-test, and differences between multiple groups were analyzed by one-way/two-way ANOVA followed by Tukey’s post hoc test. The data are shown as the mean ± SD, and *p* < 0.05 was considered significant.

**
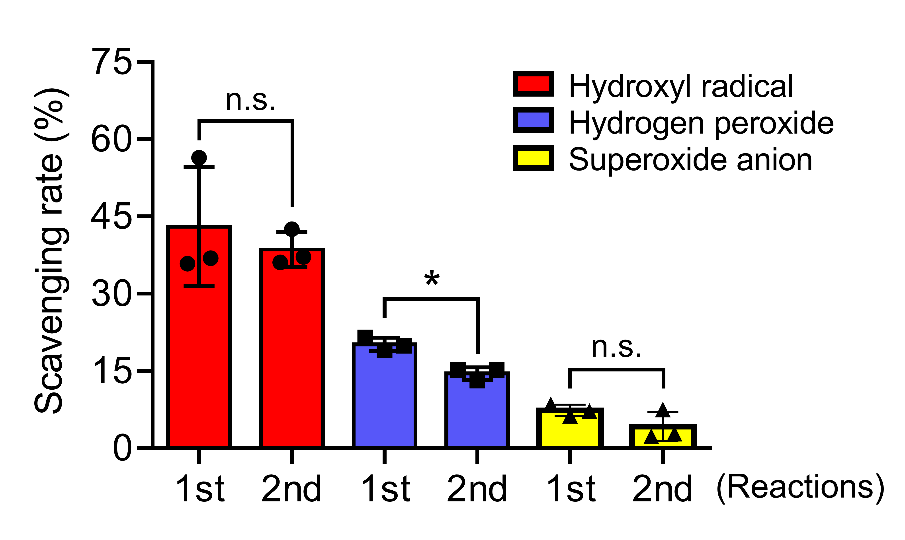
**

**Figure S1. Enzyme activity evaluation of Pd@insulin nanoclusters for multiple ROS scavenging ability.**

Enzyme activity evaluation of Pd@insulin nanoclusters for superoxide anion, hydrogen peroxide, and hydroxyl radical scavenging ability. Data are all shown as mean ± SD. Statistical analysis was performed by two-way *t*-test. n.s. denotes no significance. * denotes *p* < 0.05.

**
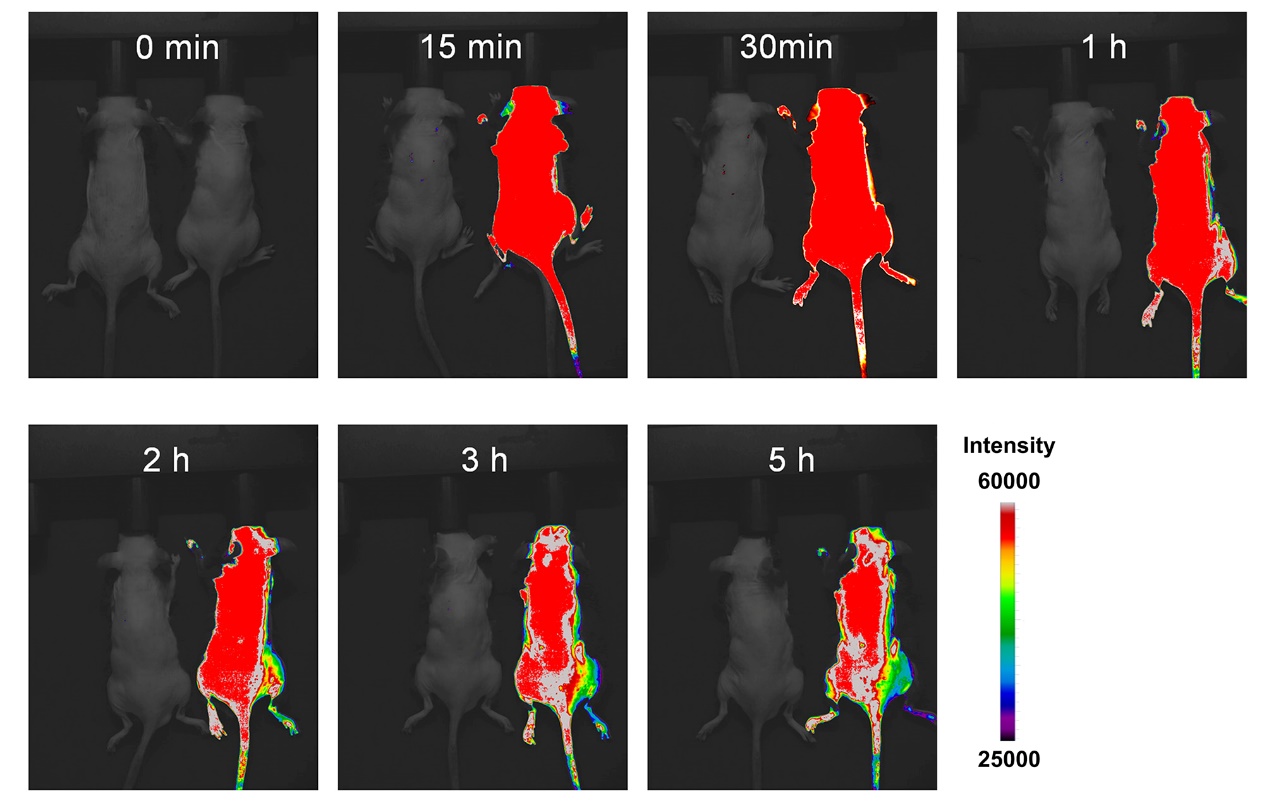
**

**Figure S2. The body distribution of Pd@insulin in 5×FAD mice post intravenous administration.**

*In vivo* fluorescence images of AD mouse treated with insulin (left) or Pd@insulin (right) at tested time points.

**Figure S3. The blood concentration of Pd@insulin in mice.**

The blood concentration of Pd@insulin was measured by the Pd^2+^ concentration. Data are all shown as mean ± SD.


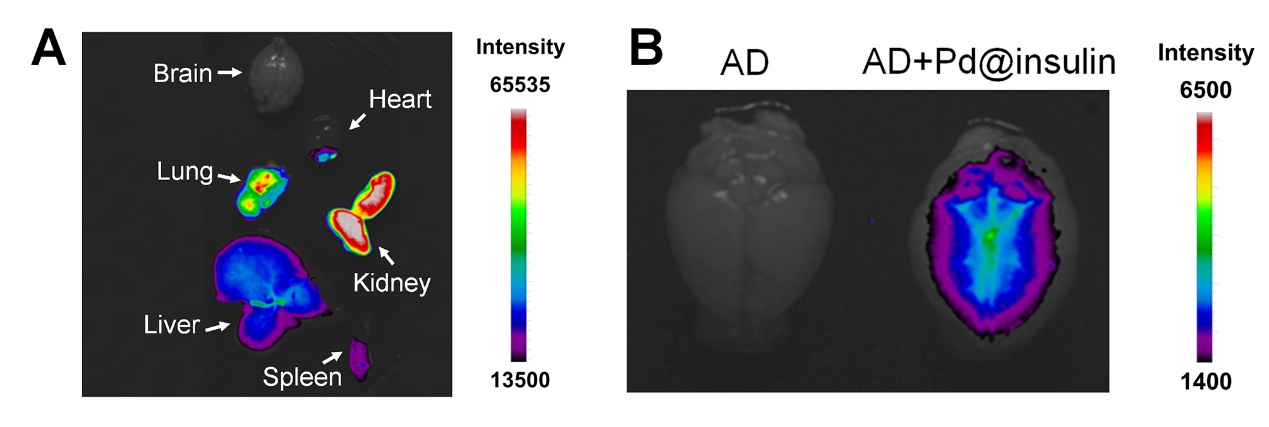


**Figure S4. The distribution of Pd@insulin in different organs of 5×FAD mice post intravenous administration.**

(**A**) Fluorescence images of different organs 30 min post Pd@insulin administration. (**B**) Fluorescence images of brains 30 min post Pd@insulin administration.


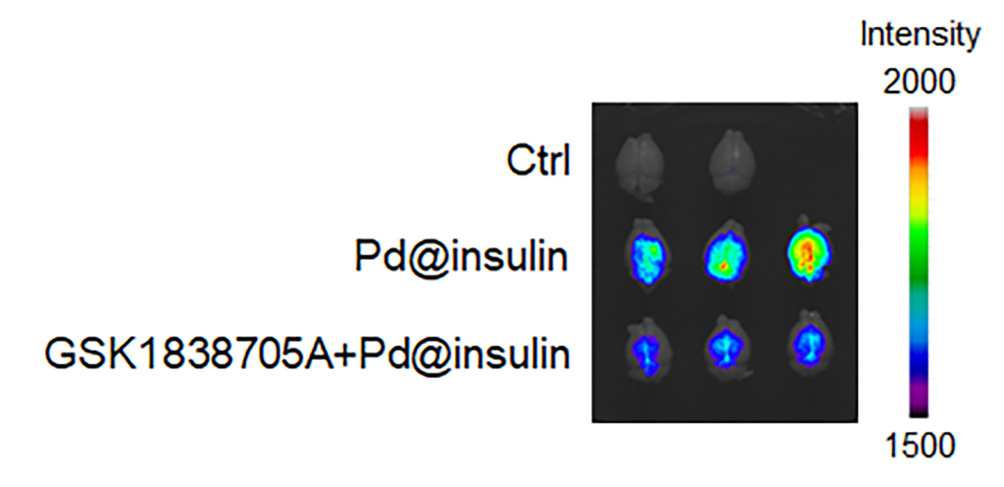


**Figure S5. The BBB crossing capacity of Pd@insulin is insulin receptor dependent.**

Fluorescence images of brains 30 min post Pd@insulin intravenous administration and GSK1838705A intraperitoneal injection (n = 3).


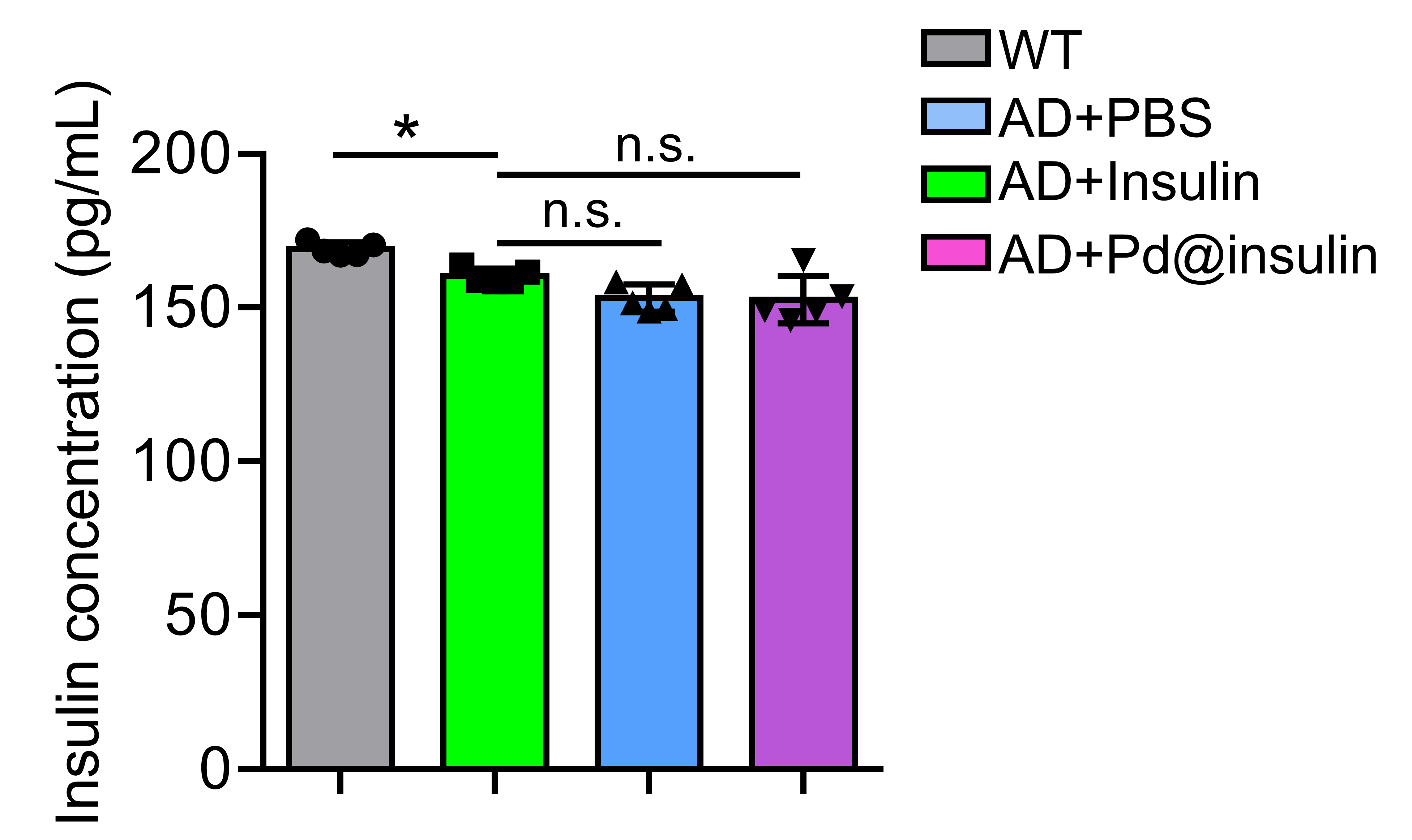


**Figure S6. The mouse brain insulin concentration 30 min post Pd@insulin intravenous administration.**

The mouse brain insulin concentration was measured 30 min post Pd@insulin or insulin intravenous administration (n = 5). Data are all shown as mean ± SD. The statistical difference among groups was assessed with the parametric one-way ANOVA with post-hoc Bonferroni test. n.s. denotes no significance. * denotes *p* < 0.05.

**Figure S7. The mouse blood glucose levels 15 min post Pd@insulin intravenous administration.**

The mouse blood glucose levels were measured with a commercial glucometer 15 min post Pd@insulin or insulin intravenous administration (n = 4). Data are all shown as mean ± SD. The statistical difference among groups was assessed with the parametric one-way ANOVA with post-hoc Bonferroni test. n.s. denotes no significance. * and *** denote p < 0.05 and p < 0.001, respectively.


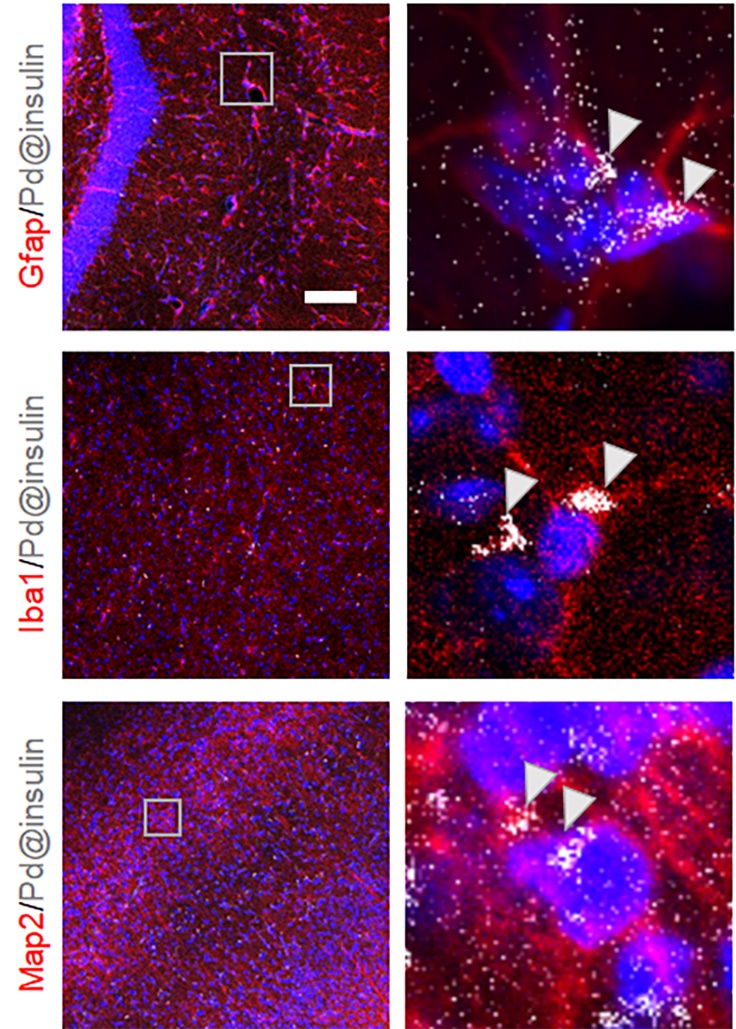


**Figure S8. The cellular distribution of Pd@insulin in the brain of 5×FAD mice post intravenous administration.**

Immunofluorescent staining of 5×FAD mice cortex (Map2, Iba1, and Gfap on behalf of neuron, microglia, and astrocyte, respectively) 30 min post intravenous administration of Cy5-NHS ester-labeled Pd@insulin nanoclusters (Scale bar 100 μm). White arrows indicate Cy5^+^ Pd@insulin nanoclusters within cells.


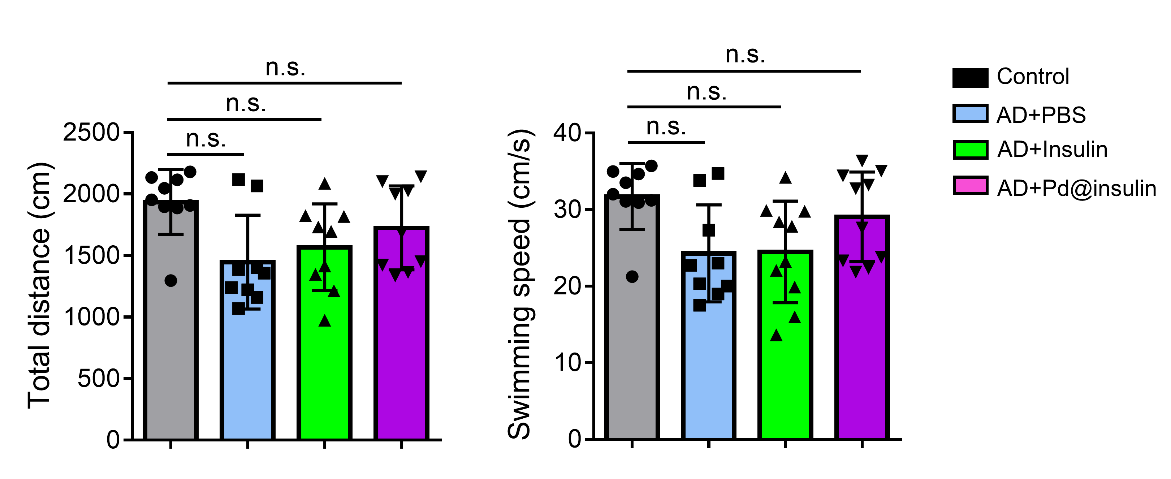


**Figure S9. Pd@insulin nanoclusters have no effects on the motor skill of 5×FAD mice in Morris Water Maze (MWM).**

Total distance and average speed of mice in MWM. n = 9~10. Scale bar = 200 μm. Data are all shown as mean ± SD. The statistical difference among groups was assessed with the parametric one-way ANOVA with post-hoc Bonferroni test. n.s. denotes no significance.


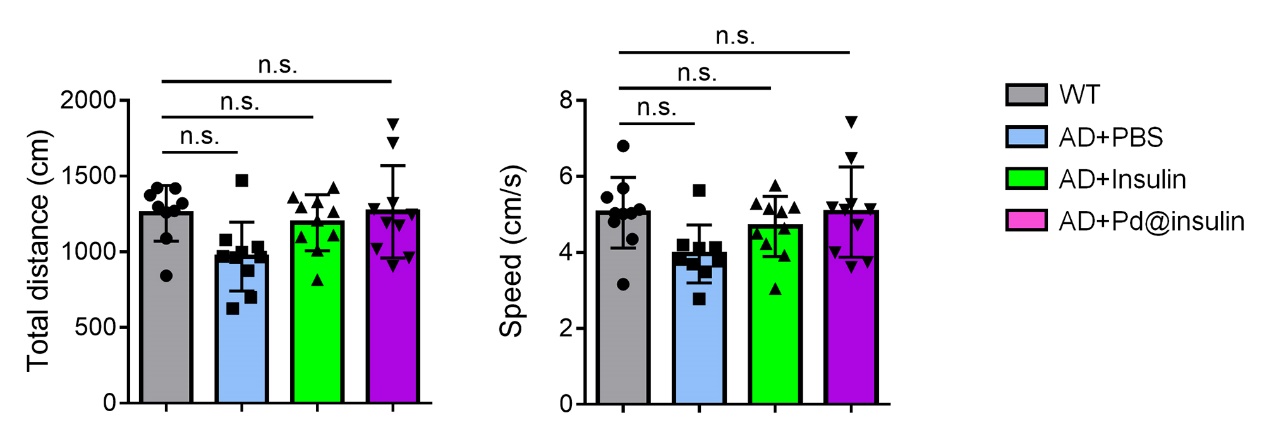


**Figure S10. Pd@insulin nanoclusters have no effects on the motor skill of 5×FAD mice in open field test (OFT).**

Total distance and average speed of mice in OFT. n = 9~10. Scale bar = 200 μm. Data are all shown as mean ± SD. The statistical difference among groups was assessed with the parametric one-way ANOVA with post-hoc Bonferroni test. n.s. denotes no significance.

**
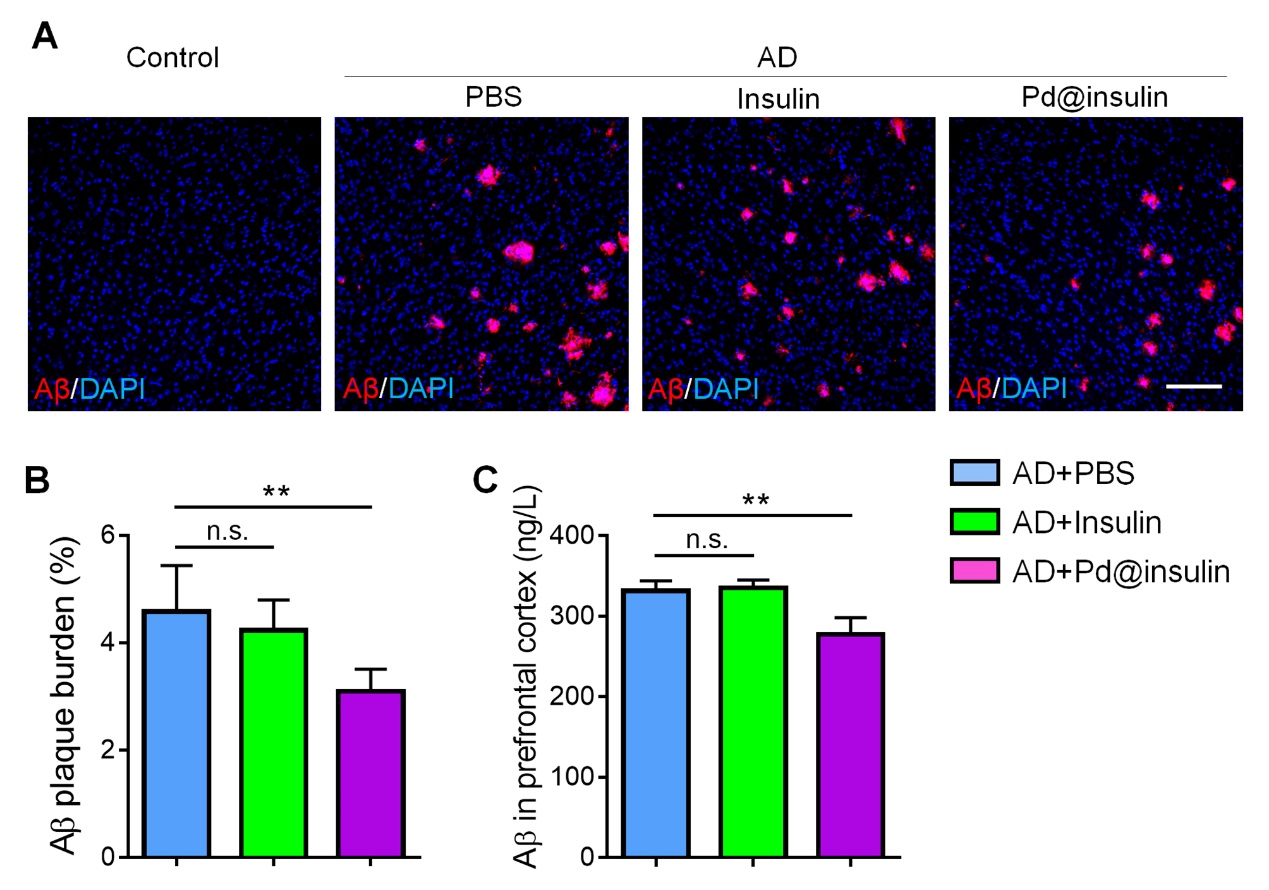
**

**Figure S11. Pd@insulin nanoclusters mitigates Aβ plaque deposition in 5×FAD mouse prefrontal cortex.**

(**A**) Representative images of Aβ immunoreactivity in the prefrontal cortex at ×20 magnification. (**B**) Aβ plaque burden was quantified using ImageJ. (**C**) The levels of Aβ_1-42_ in the prefrontal cortex were determined by ELISA assay. n = 4. Scale bar = 200 μm. Data are all shown as mean ± SD. The statistical difference among groups was assessed with the parametric one-way ANOVA with post-hoc Bonferroni test. n.s. denotes no significance. ** denotes *p* < 0.01.


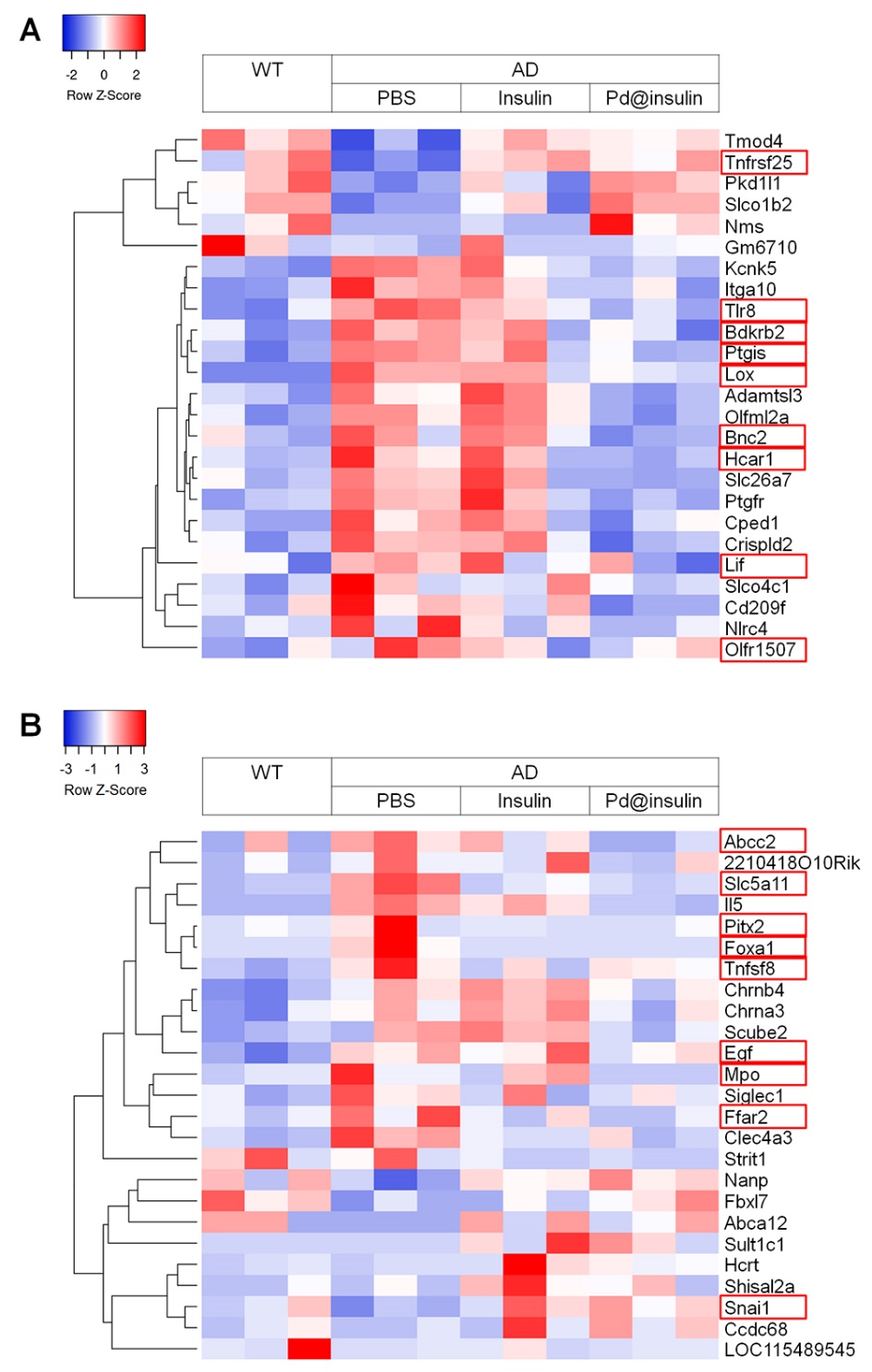


**Figure S12.** **Pd@insulin alters gene expression profiles in 5×FAD mouse** **hippocampal and prefrontal cortical tissues.**

(**A**) Heatmap of top 25 DEGs in 5×FAD mouse hippocampal tissues among groups in comparison between Pd@insulin- and PBS- inject mice. (**B**) Heatmap of top 25 DEGs in 5×FAD mouse prefrontal cortical tissues among groups in comparison between Pd@insulin- and PBS- inject mice. Red boxes indicate ROS-related genes reported by published studies.


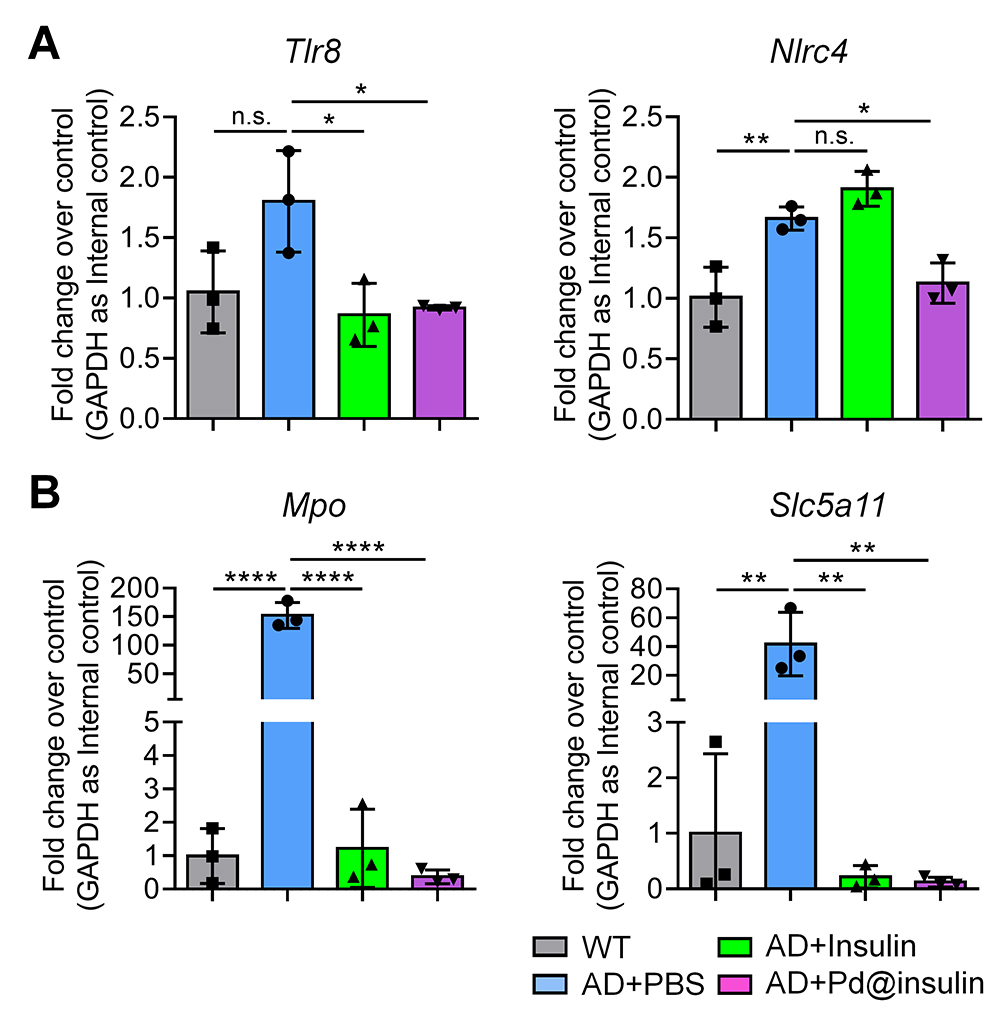


**Figure S13.** **qRT-PCR validation for RNA-seq analysis.**

(**A**) qRT-PCR analysis of the expression levels of transcripts corresponding to *Tlr8* and *Nlrc4* among groups in comparison between the hippocampal tissues of Pd@insulin- and PBS- inject mice. (**B**) qRT-PCR analysis of the expression levels of transcripts corresponding to *Mpo* and *Slc5a11* among groups in comparison between the prefrontal cortical tissues of Pd@insulin- and PBS- inject mice. n = 3. Data are all shown as mean ± SD. The statistical difference among groups was assessed with the parametric one-way ANOVA with post-hoc Bonferroni test. n.s. denotes no significance. *, **, and **** denote *p* < 0.05, *p* < 0.01, and *p* < 0.0001, respectively.


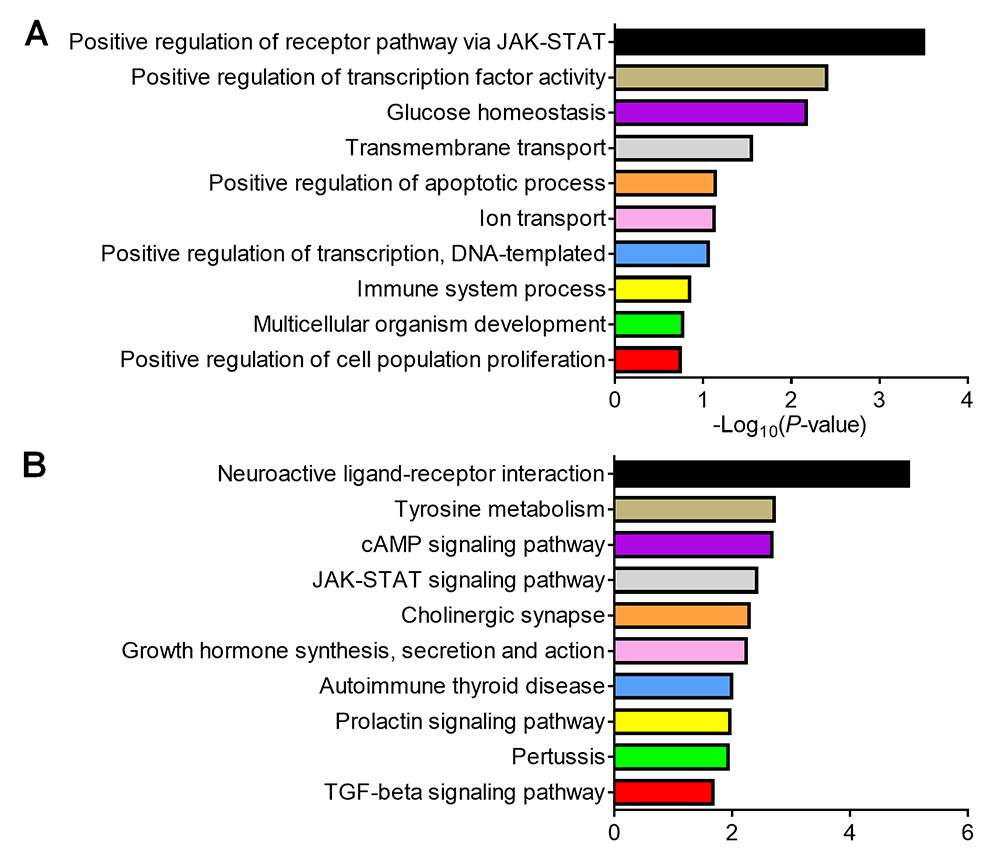


**Figure S14.** **Bioinformatic analyses for DEGs in comparison between prefrontal cortical tissues of Pd@insulin- and PBS- injected mice.**

(**A**) The top 10 GO terms of DEGs in comparison between Pd@insulin- and PBS- inject mice. (**B**) The top 10 KEGG pathways of DEGs in comparison between Pd@insulin- and PBS- inject mice.


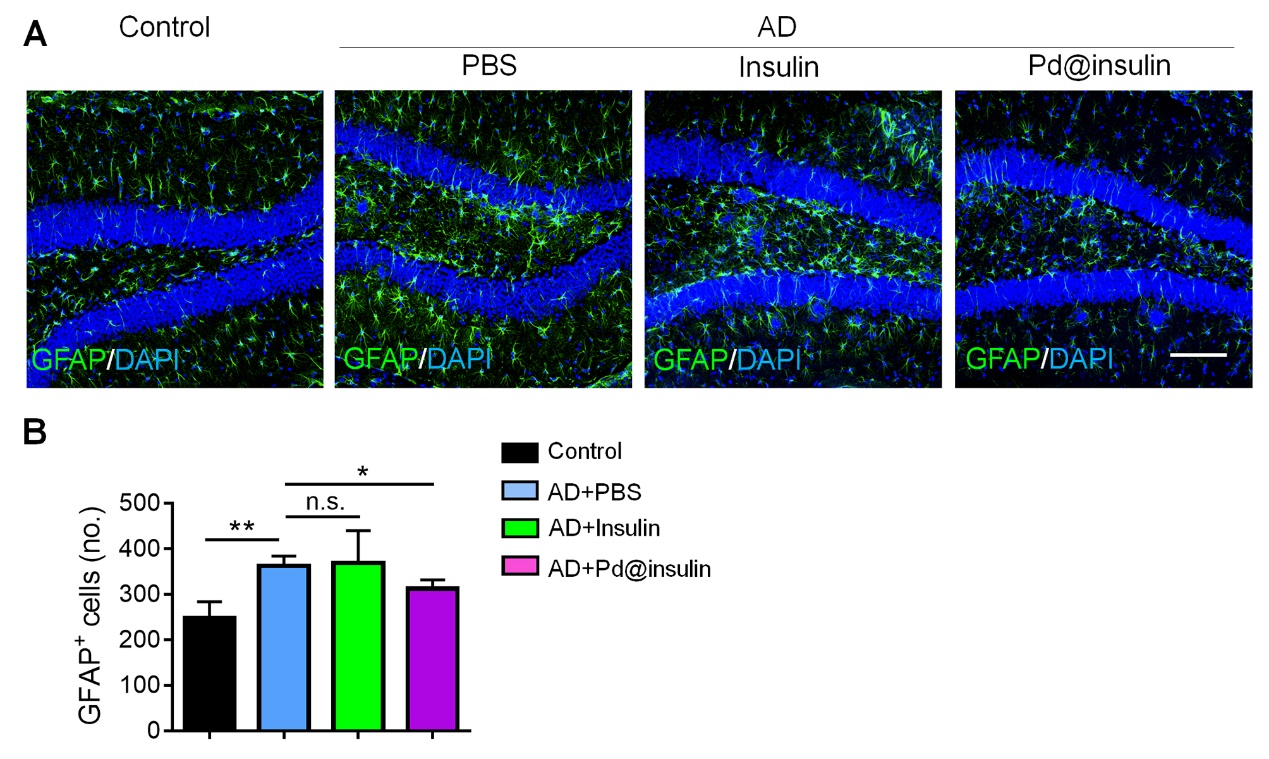


**Figure S15.** **Pd@insulin inhibits astrocyte activation in 5×FAD mouse hippocampus.**

(**A**) Representative confocal microscopy images of GFAP immunoreactivity in the hippocampus at ×20 magnification. (**B**) Numbers of immunoreactive cells in each group were quantified by ImageJ (n = 4). Scale bar: 200 μm. Data are all shown as mean ± SD. n.s. denotes no significance. * and ** denote *p* < 0.05 and *p* < 0.01, respectively. The statistical difference among groups was assessed with the parametric one-way ANOVA with post-hoc Bonferroni test.


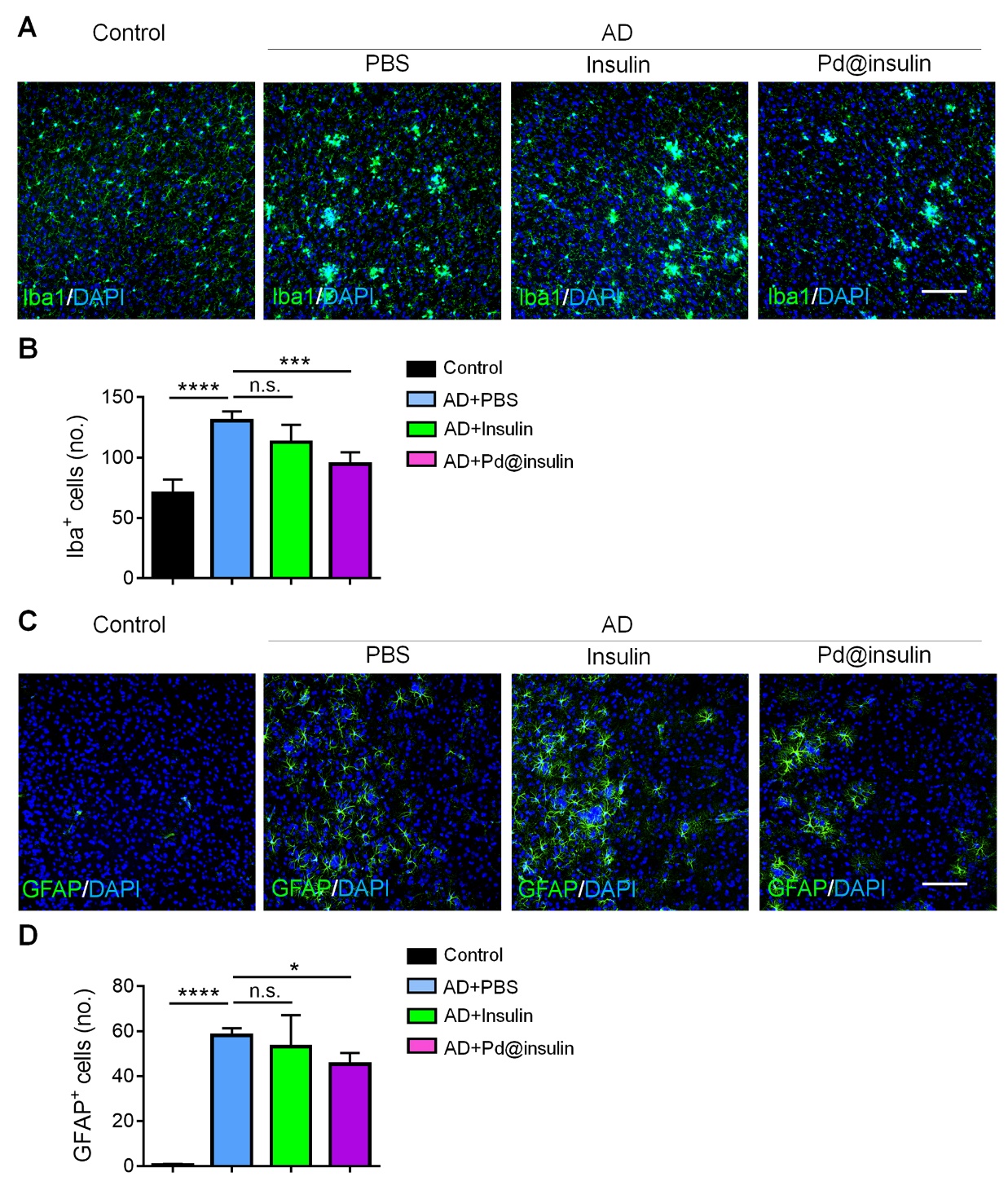


**Figure S16.** **Pd@insulin inhibits glial activation in 5×FAD mouse prefrontal cortex.**

(**A**) Representative confocal microscopy images of Iba1 immunoreactivity in the prefrontal cortex at ×20 magnification. (**B**) Numbers of Iba1^+^ cells in each group were quantified by ImageJ (n = 4). (**C**) Representative confocal microscopy images of GFAP immunoreactivity in the prefrontal cortex at ×20 magnification. (**D**) Numbers of GFAP^+^ cells in each group were quantified by ImageJ (n = 4). Scale bar: 200 μm. Data are all shown as mean ± SD. n.s. denotes no significance. *, ***, **** denote *p* < 0.05, *p* < 0.001, *p* < 0.0001, respectively. The statistical difference among groups was assessed with the parametric one-way ANOVA with post-hoc Bonferroni test.


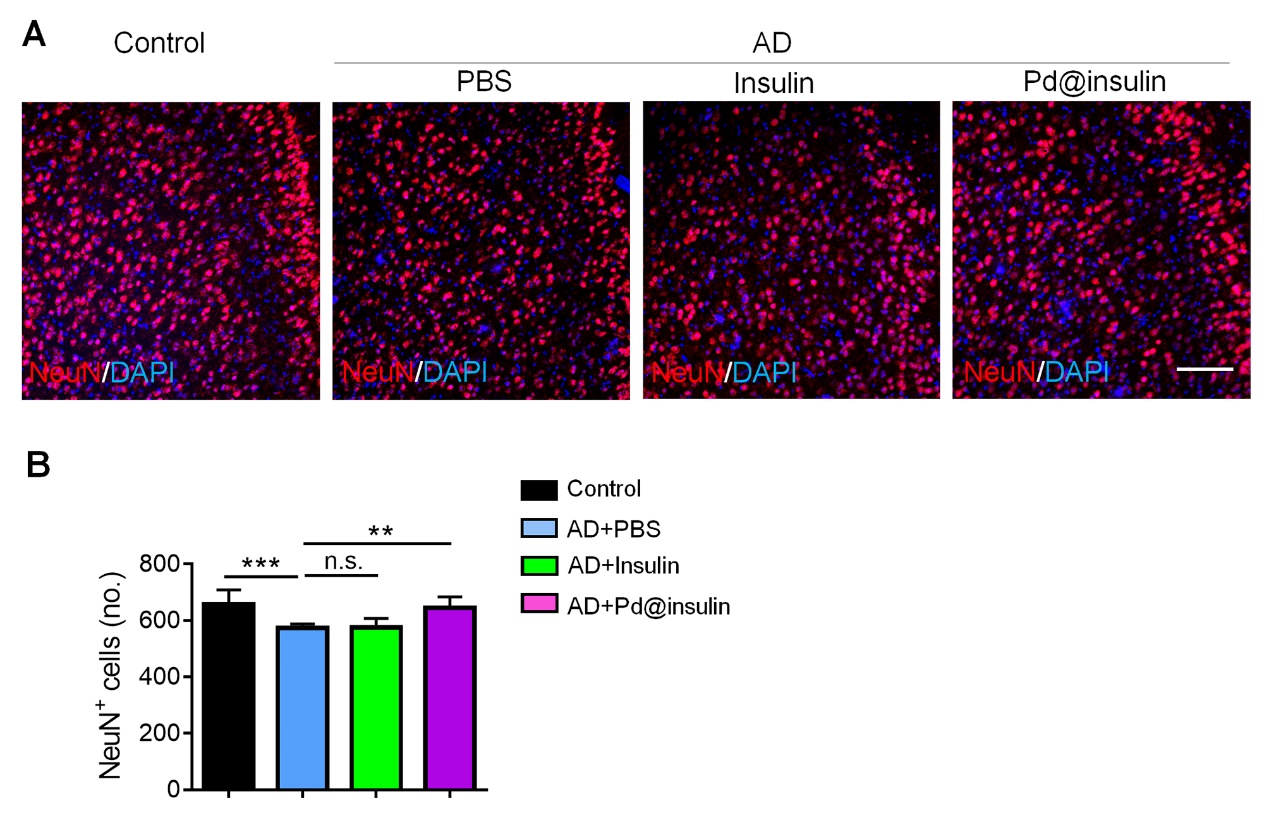


**Figure S17.** **Pd@insulin inhibits neuronal loss in 5×FAD mouse prefrontal cortex.**

(**A**) Representative confocal microscopy images of NeuN immunoreactivity in the prefrontal cortex at ×20 magnification. (**B**) Numbers of NeuN^+^ cells in each group were quantified by ImageJ (n = 4). Scale bar: 200 μm. Data are all shown as mean ± SD. n.s. denotes no significance. ** and *** denote *p* < 0.01 and *p* < 0.001, respectively. The statistical difference among groups was assessed with the parametric one-way ANOVA with post-hoc Bonferroni test.


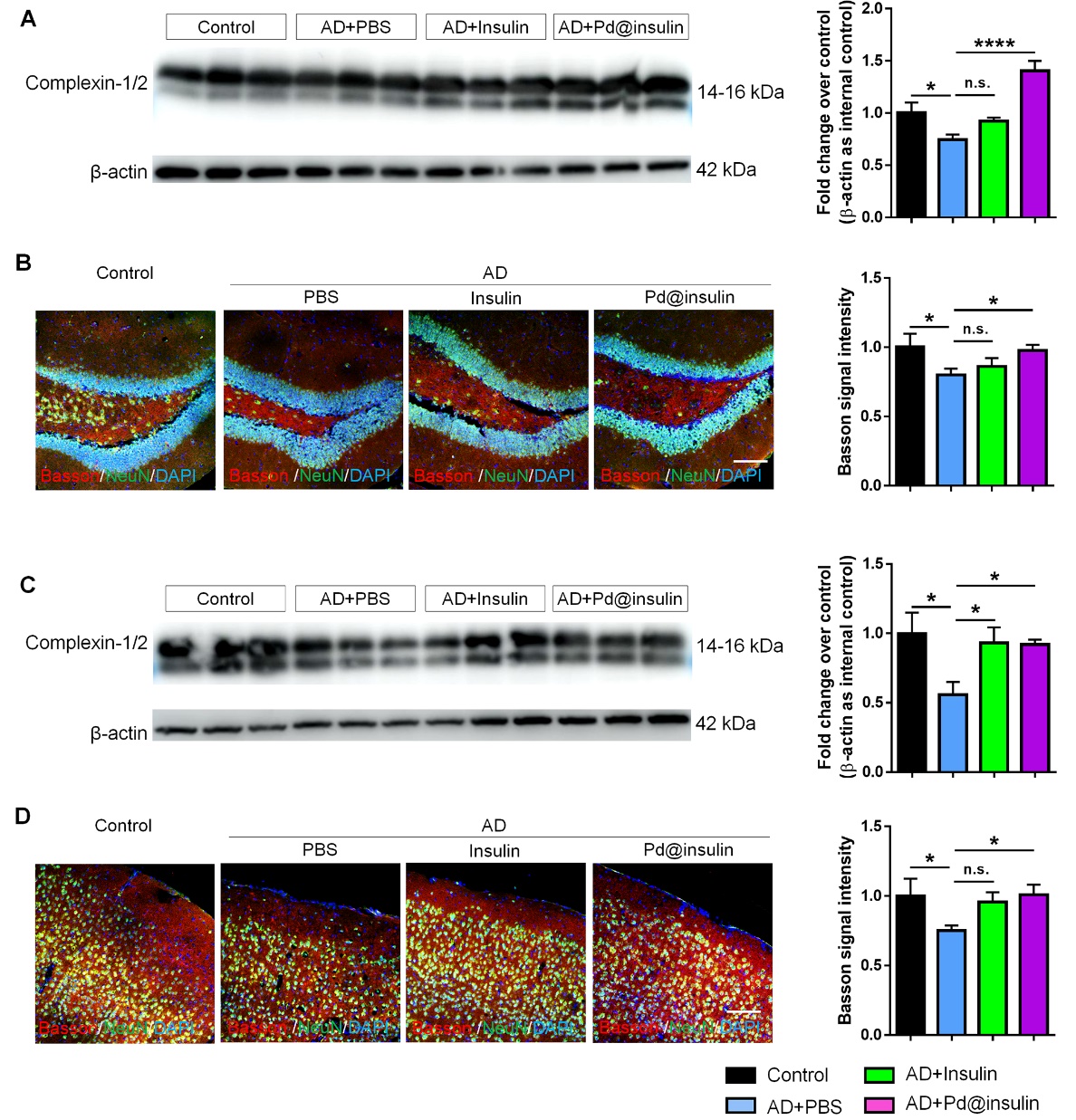


**Figure S18.** **Pd@insulin inhibits synaptic damage in 5×FAD mouse brains.**

(**A**) Representative blots (left) and quantification (right) of Complexin-1/2 expression in the hippocampus (n = 3). (**B**) Representative confocal microscopy images of Basson (red) and NeuN (green) immunoreactivity in the hippocampus at ×20 magnification. Basson signal intensities were quantified by ImageJ (n = 3). (**C**) Representative blots (left) and quantification (right) of Complexin-1/2 expression in the prefrontal cortex (n = 3). (**D**) Representative confocal microscopy images of Basson (red) and NeuN (green) immunoreactivity in the prefrontal cortex at ×20 magnification. Basson signal intensities were quantified by ImageJ (n = 3). Scale bar: 200 μm. Data are all shown as mean ± SD. n.s. denotes no significance. ** and *** denote *p* < 0.01 and *p* < 0.001, respectively. The statistical difference among groups was assessed with the parametric one-way ANOVA with post-hoc Bonferroni test.


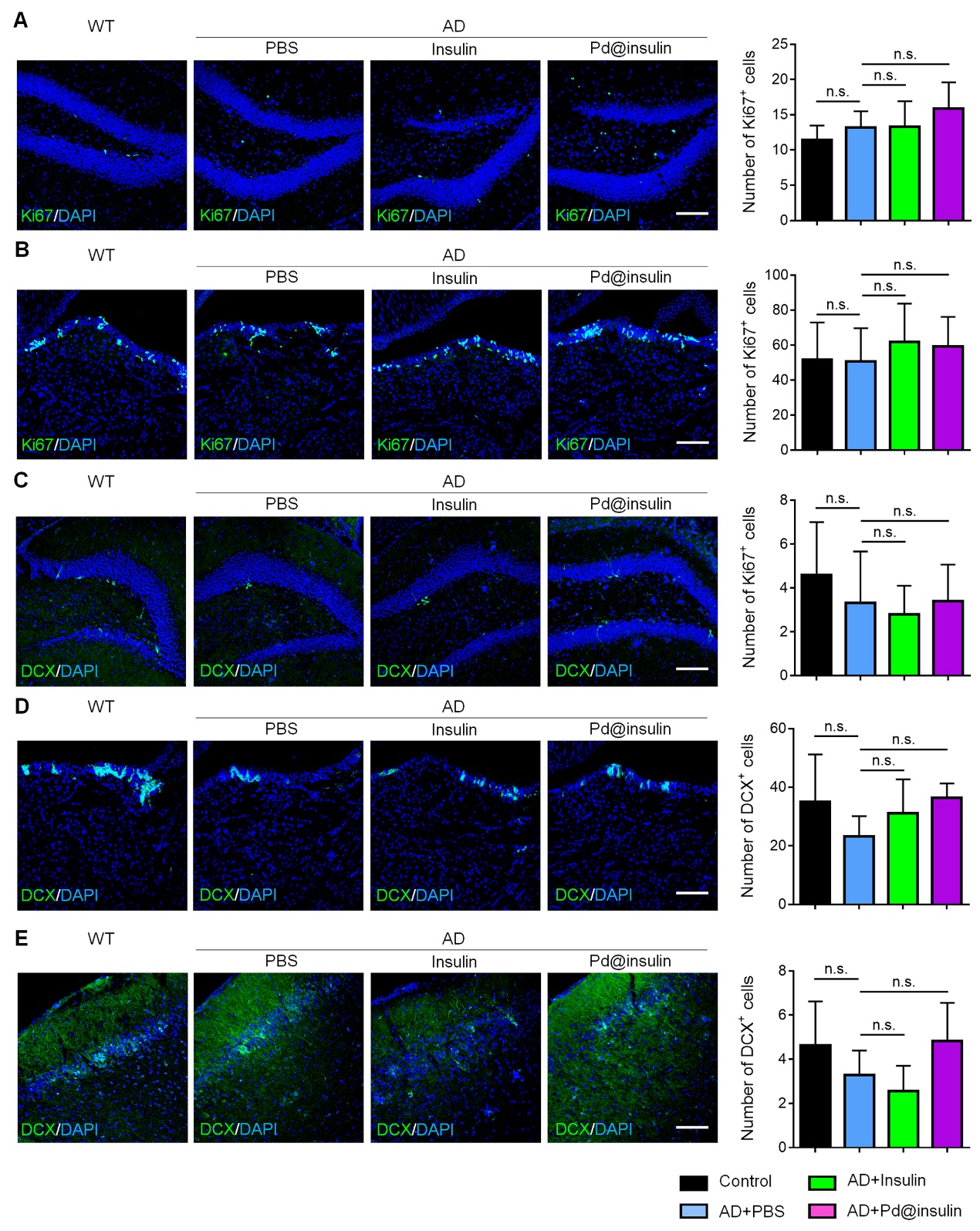


**Figure S19.** **Pd@insulin has no effect on neurogenesis in 5×FAD mouse brain.**

(**A-B**) Representative confocal microscopy images of Ki67 immunoreactivity in the hippocampus (**A**) and subventricular zone (**B**) at ×20 magnification. Numbers of Ki67^+^ cells in each group were given in the right panel (n = 4). (**C-E**) Representative confocal microscopy images of DCX immunoreactivity in the hippocampus (**C**), subventricular zone (**D**), and prefrontal cortex (**E**) at ×20 magnification. Numbers of GFAP^+^ cells in each group were given in the right panel (n = 4). Scale bar: 200 μm. Data are all shown as mean ± SD. n.s. denotes no significance. The statistical difference among groups was assessed with the parametric one-way ANOVA with post-hoc Bonferroni test.
